# News and Miscellany

**Published:** 1904-03

**Authors:** 


### News and Miscellany.

---

Dr. W. B. McLaughlin, of Austin, read a paper before the New York Academy of Medicine, February 18th (ult.), on "The Rationale of Natural Arrest of Consumption; a Study Explaining the Effect of Sea Voyages, Out-of-Doors Life, etc., on the Disease, with Suggestions as to Ideal Treatment." It was discussed by Drs. Peabody, W. Gilman Thompson, Jno. B. Huber, and Major W. C. Gorgas, Surgeon, U. S. A. Major Gorgas had charge of the sanitary reform in Havana, which led to the extinguishment of yellow fever, and Dr. McLaughlin was associated with him in the work.

---

As to Dr. Knopf.—Dr. Knopf wrote me that I had been misinformed, and that I had no warrant for saying that his letter (see February number Texas Medical Journal), in which he asked the conditions of membership in the International Congress of Tuberculosis, was not answered. He sends me a letter from Dr. Barrick, the President of the Congress, which, he says, is an answer to his letter, and asks me to publish it. I do so with pleasure. It will be seen that Dr. Barrick is "glad to know that he (Dr. Knopf) is interested in the great humanitarian movement," and refers the letter to the Chairman of the Organization Committee (also Chairman of the Executive Council). The case is not the least altered by this letter, for the chairman did not answer it, and I was officially notified that the Executive Committee unanimously "resolved that it was not to the interest of the Congress to admit applicant to membership;" just as I stated. Here is the letter, however, which Dr. K. calls a reply, and asks me to publish. See for yourself what it amounts to:

(Copy.)

American Congress on Tuberculosis.

Season of 1903-4.

Office of the President.

Toronto, Ontario, November 16, 1903.

*S. A. Knopf, M. D., 16 West Ninety-fifth Street, New York.*

My Dear Doctor: I am in receipt of your letter of the 11th inst., and am glad to know that your interest in the great humanitarian movement to prevent the spread of tuberculosis is still keeping up.

As I am not in possession of all the information you ask for, I have forwarded your letter to the Chairman of our Executive Committee, who is also Chairman of the Committee of Organization of the International Congress on Tuberculosis, appointed by the President of the Universal Exposition; I refer to Mr. Clark Bell, 39 Broadway, New York.

Under separate cover I sent to your address a pamphlet in support of Municipal Sanatoria for Consumptives. I shall be obliged if you would read it carefully and write me how it appeals to you.

Yours very truly,

E. J. Barrick.

---

See Here, Doctor:

| | |
|---|---|
| "Red Back," one year | $1 00 |
| *Daily Medical Journal,* New York | 1 00 |
| Both sent for | $1 50 |

---

Belated Yellow Fever.—Two cases of yellow fever occurred in Laredo in January; one on the 2d and one on the 5th.

---

Dr. J. H. Foster, Second Assistant Physician at the State Lunatic Asylum, has resigned; Dr. Louis H. Kirk, Third Assistant, has been promoted to the vacancy, and Dr. John Bradfield, of Daingerfield, has been appointed third assistant, and Dr. Horace Gilbert, of Austin, has been appointed fourth assistant. These young men are all graduates of the Medical Department, Texas University.

---

Notice to Delegates, Texas State Medical Association Meeting, at Austin in April.—Delegates will meet at 2 o'clock, April 25th (one day ahead of meeting of the Association), to organize the House of Delegates.

---

### In Memoriam—Dr. H. A. West.

*To the President and Members of the Fifth District Medical Association:*

We, your committee appointed to draft resolutions upon the death of Drs. H. A. West, of Galveston, and J. C. Jones, of Gonzales, beg leave to report that:

Death has invaded our rank and claimed two of the most distinguished members of the medical profession of Texas; the gallant Jones and the companionable West; the one retiring and modest as a woman and the other the eloquent debator and champion of all that goes to upbuild the medical profession, but both the educated, respected and lovable physicians, honored and respected by all the profession of the State. Dr. Jones was a distinguished surgeon in the Confederate army, and the physician to the great commander, General Hood. He was highly educated in the great universities of Eupore, and served his State honorably as a member of the Board of Medical Examiners for the State of Texas from its organization until his death. Dr. West was a distinguished medical teacher and physician, who gave a large portion of his time to the upbuilding of the State Medical Association, having served as its Secretary for the past ten years. Therefore, be it

*Resolved,* By the President and Members of the Fifth District Medical Association, in session at San Antonio, Texas, February 4, 1904, That we wish to express our sorrow for the loss of these men who were untiring in their efforts to elevate the standard of medicine in Texas, and who exemplified in their lives the noblest qualities of mind and heart of the true Medical Practitioner. That we express to their respective families our deepest sympathy in their bereavement. That a copy of these resolutions be sent to their families and given for publication to the press of the State.

L. L. SHROPSHIRE,
M. M. SMITH,
A. GARWOOD,
T. T. JACKSON,
M. T. MOORE.

---

*To Our Texas Friends, Through the "Red Back":*

It gives us much pleasure to announce that, while the recent fire at Baltimore threatened our laboratories, we were fortunate to escape without loss, and are still doing business at the old corner. We are perfectly able to take care of and ship any order with which our friends may favor us.

Very truly yours,

SHARP & DOHME.

[Try their Glycero Phosphates; a powerful and elegant reconstructive tonic.—DANIEL.]

---

$3000 CASH.—Office practice free to purchaser of fine office equipment; at great bargain. Good location. Buyer introduced. Write promptly. "Doctor," Box 395, Paris, Texas.

---

MEETING OF THE BOARD OF MEDICAL EXAMINERS FOR THE STATE OF TEXAS.—The next meeting of the Board of Medical Examiners for the State of Texas (Regular), to examine applicants to practice medicine, surgery and midwifery in this State, will be held in Austin, Texas, April 21, 22 and 23, 1904. For further information, address Dr. M. M. Smith, Secretary, Austin, Texas.

---

Big Injun.—An elegant lithograph, in eleven colors, of a "Medicine Man of the Sioux Indians" has been sent to every physician in the United States by the proprietors of the Tongaline Preparations and Ponca Compound. Any physician who has not received this handsome and artistic reproduction of a famous Indian chief, can easily obtain such by writing for it to the Mellier Drug Company, St. Louis.

---

Those Emergency Cases.—We mentioned in our last issue that Mr. E. Hadra, of Dallas, had suggested the carrying of emergency cases on all railroads for use in case of accidents, and said that it would seem that some chief surgeon would have inaugurated such a measure long ago. Chief Surgeon S. C. Red, of the Houston & Texas Central, informs me that "such an emergency packet has been on all trains of the H. & T. C. for a very long time." I take pleasure in making the statement.

---

For Sale.—Drug store and home of a deceased physician. Large practice for capable German physician in a large German neighborhood. Former doctor's cash practice amounted to over $3000 per annum. For particulars, write to Judge S. R. Blake, Bellville, Texas.

---

Will Repair Your Electrical Machines.—Static and all electrical medical apparatus put in running order. I am also agent for electrical and X-Ray apparatus. Oliver Brush, 710 Colorado Street, Austin, Texas.

---

New Orleans Polyclinic.—Seventeenth annual session opens November 2, 1903, and closes May 28, 1904. Physicians will find the Polyclinic an excellent means for posting themselves upon modern progress in all branches of medicine and surgery. The specialties are fully taught, including laboratory work. For further information, address New Orleans Polyclinic, postoffice box 797, New Orleans, La.

---

Alkalometry in Pneumonia.—There are those who claim that nothing remedial can be done for pneumonia. The Abbott Alkaloidal Company are very sure that something can be done by Alkalometry, and they announce the claim in their advertisement this month, which please see. Dr. Abbott will be pleased to give any inquiry as to alkaloids, etc., his personal and prompt attention. Write to him.

---

An Electro-Therapeutist's Definition of a Kiss.—A gradual obliteration of the spark-gap, resulting in a short circuit while the machine works under increased pressure, the electrical energy being given off in the form of elliptical (pronounced a-lip-tickle) vibrations.—*Exchange.*

---

New York, January 25, 1904.

*Texas Medical Journal.*

Dear Sirs: Will you very kindly give space to the following notice in your valued journal, and oblige:

X-Ray Prize Essays.—Believing that the further development of X-Rays is of great importance to surgery and medicine and the human race, and to encourage research and disseminate the knowledge gained, the *Illustrated Review of Physiologic Therapeutics* offers the sum of fifteen hundred dollars in cash prizes for the best "Essays on X-Rays in Medicine and Surgery," the first prize being $1000. All surgeons, physicians and hospitals interested in any branch of X-Ray work should write to the *Illustrated Review of Physiologic Therapeutics,* 19 East Sixteenth Street, New York City, for information concerning title, time allowed, conditions, etc.

For your information, we may say that we give this prize on about the same plan as the recent "Preventive Medicine" prizes.

Very truly yours,

Illustrated Review of Physiologic Therapeutics,

19 East Sixteenth Street.

